# Multidisciplinary, Clinical Assessment of Accelerated Deep-Learning MRI Protocols at 1.5 T and 3 T After Intracranial Tumor Surgery and Their Influence on Residual Tumor Perception

**DOI:** 10.3390/diagnostics15151982

**Published:** 2025-08-07

**Authors:** Christer Ruff, Till-Karsten Hauser, Constantin Roder, Daniel Feucht, Paula Bombach, Leonie Zerweck, Deborah Staber, Frank Paulsen, Ulrike Ernemann, Georg Gohla

**Affiliations:** 1 Department of Diagnostic and Interventional Neuroradiology, Eberhard Karls-University Tübingen, D-72076 Tübingen, Germany; 2 Department of Neurosurgery, University of Tuebingen, D-72076 Tuebingen, Germany; 3 Center for Neuro-Oncology, Comprehensive Cancer Center Tuebingen-Stuttgart, University Hospital of Tuebingen, Eberhard Karls University of Tuebingen, D-72070 Tuebingen, Germany; 4 Department of Neurology and Interdisciplinary Neuro-Oncology, University Hospital Tuebingen, D-72076 Tuebingen, Germany; 5 Hertie Institute for Clinical Brain Research, Center of Neuro-Oncology, Eberhard Karls University Tuebingen, D-72076 Tuebingen, Germany; 6 Department of Radiation Oncology, University Hospital Tuebingen, D-72076 Tuebingen, Germany

**Keywords:** intracranial tumors, postoperative imaging, magnetic resonance imaging, deep learning, image reconstruction, multidisciplinary, diagnostic accuracy, visual perception preference

## Abstract

**Background/Objectives**: Postoperative MRI is crucial for detecting residual tumor, identifying complications, and planning subsequent therapy. This study evaluates accelerated deep learning reconstruction (DLR) versus standard clinical protocols for early postoperative MRI following tumor resection. **Methods**: This study uses a multidisciplinary approach involving a neuroradiologist, neurosurgeon, neuro-oncologist, and radiotherapist to evaluate qualitative aspects using a 5-point Likert scale, the preferred reconstruction variant and potential residual tumor of DLR and conventional reconstruction (CR) of FLAIR, T1-weighted non-contrast and contrast-enhanced (T1), and coronal T2-weighted (T2) sequences for 1.5 and 3 T MRI. Quantitative analysis included the image quality metrics Structural Similarity Index (SSIM), Multi-Scale SSIM (MS-SSIM), Feature Similarity Index (FSIM), Noise Quality Metric (NQM), signal-to-noise ratio (SNR), and Peak SNR (PSNR) with CR as a reference. **Results**: All raters strongly preferred DLR over CR. This was most pronounced for FLAIR images at 1.5 and 3 T (91% at 1.5 T and 97% at 3 T) and least pronounced for T1 at 1.5 T (79% for non-contrast-enhanced and 84% for contrast-enhanced sequences) and for T2 at 3 T (69%). DLR demonstrated superior qualitative image quality for all sequences and field strengths (*p* < 0.001), except for T2 at 3 T, which was observed across all raters (*p* = 0.670). Diagnostic confidence was similar at 3 T with better but non-significant differences for T2 (*p* = 0.134) and at 1.5 T with better but non-significant differences for non-contrast-enhanced T1 (*p* = 0.083) and only marginally significant results for FLAIR (*p* = 0.033). Both the SSIM and MS-SSIM indicated near-perfect similarity between CR and DLR. FSIM performs worse in terms of consistency between CR and DLR. The image quality metrics NQM, SNR, and PSNR showed better results for DLR. Visual assessment of residual tumor was similar at 3 T but differed at 1.5 T, with more residual tumor detected with DLR, especially by the neurosurgeon (*n* = 4). **Conclusions**: An accelerated DLR protocol demonstrates clinical feasibility, enabling high-quality reconstructions in challenging postoperative MRIs. DLR sequences received strong multidisciplinary preference, underscoring their potential to improve neuro-oncologic decision making and suitability for clinical implementation.

## 1. Introduction

Postoperative imaging following intracranial tumor resection is standard for evaluating residual disease, detecting complications, and planning further treatment [1,2,3,4,5]. A comprehensive MRI protocol should include both non-enhanced and contrast-enhanced T1-weighted and T2-weighted sequences, as well as fluid-attenuated inversion recovery (FLAIR) imaging [6]. Misclassification postoperatively can lead to an inaccurate assessment of tumor progression or treatment response in follow-up examinations. Consequently, precise visualization and recognition of residual tumor are imperative to distinguish postoperative changes from residual tumor areas in subsequent scans. Postoperative MRI is challenging. Air–tissue interfaces, metallic implants, and tissue distortion create susceptibility artifacts, especially on conventional EPI-based diffusion-weighted imaging (DWI) sequences. Moreover, the mental state of tumor patients who have undergone surgery is diminished, thereby emphasizing the necessity for quick diagnostic examinations. Prolonged scan times can result in physical discomfort for the patient and, therefore, introduce image motion artifacts or even premature termination of the examination [7].

The trade-off between high-resolution imaging and scan time limits conventional MRI techniques. A critical consideration is achieving the optimal balance between the three interdependent imaging parameters: image resolution, signal-to-noise ratio (SNR), and acquisition time. While higher resolution enables the visualization of finer details, it is often accompanied by a reduction in signal-to-noise ratio (SNR) and/or an increase in acquisition time [8]. Concurrently, a minimum level of SNR is imperative to discern the signal of interest from system noise. Accurate delineation of tumor boundaries, a crucial element for effective treatment planning and monitoring, can be compromised by image quality issues.

Artificial intelligence (AI) applications in medical imaging have garnered significant attention, as they hold considerable promise in overcoming the obstacles mentioned above [9]. This has resulted in substantial advancements in image quality and the integration of neural network technologies, including neuro-oncological imaging [7,10]. In particular, deep learning (DL) has emerged as a crucial technique to overcome the limitations of traditional acceleration methods in imaging. State-of-the-art DL models are designed to enhance clinical diagnosis by improving accuracy, speed, and overall capabilities [11]. Models such as deep neural networks, variational autoencoders, and generative adversarial networks have been demonstrated to accelerate MRI acquisition, reduce patient discomfort, and enhance workflow [11]. Convolutional neural networks with attention mechanisms and U-Net architectures can improve MRI spatial resolution and thereby enhance anatomical visualization. AI has shown considerable promise in various applications in brain tumor management, encompassing diagnostic imaging, prognostication, and treatment planning. This potential extends beyond the conventional uses of AI, such as MRI acceleration and enhancement [12,13]. Integrating AI into radiology and pathology workflows can advance neuro-oncology. In glioblastoma and IDH-mutant gliomas, deep learning algorithms have reduced acquisition times by approximately 30% and improved subjective image quality [7,10,14,15].

A recent study evaluated an experimental approach to employing DL reconstruction (DLR) for intraoperative MRI in patients under general anesthesia [16]. However, currently, there is a lack of research on the use of DLR in the postoperative setting. It is essential to recognize the role of expert groups in developing consensus-based guidelines for scheduled postoperative surveillance MRI. Organizations such as the National Institute of Health and Care Excellence (NICE, postoperative scan < 72 h) and the European Association of Neuro-Oncology (EANO, postoperative scan < 48 h) provide timing recommendations for postoperative MRI to confirm the extent of resection or residual enhancing tumor [17]. This time window is recommended to be observed postoperatively to avert the occurrence of nonneoplastic contrast enhancement due to surgical manipulation [18,19].

A multidisciplinary review of postoperative imaging using a DL protocol has not yet been reported. DLR could shorten scan times, improve image quality, and alter residual-disease assessment by changing image appearance and increasing SNR in vulnerable postoperative patients. Therefore, this study compared an accelerated DL protocol with our conventional 1.5 Tesla (T) and 3 T reconstructions. We evaluated whether DLR facilitates the detection of residual tumors after intracranial tumor resection in a multidisciplinary setting.

## 2. Materials and Methods

### 2.1. Study Design

**Figure 1:**
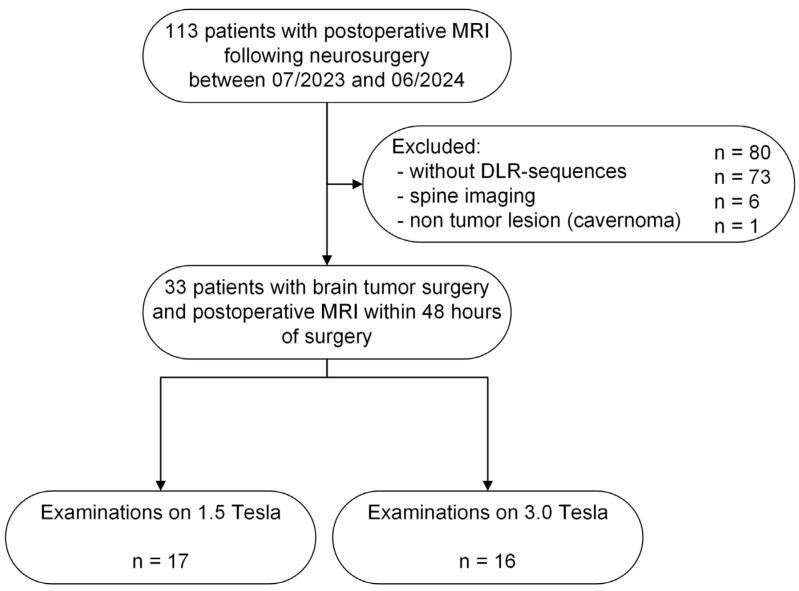
Study flowchart and patient enrollment.

The institutional review board approved this monocentric retrospective study under code 549/2024BO2. All study procedures followed the Declaration of Helsinki and its subsequent amendments. This study comprised 33 patients who underwent cerebral MRI at 1.5 T or 3 T within the first 48 h following tumor resection for therapy assessment between July 2023 and June 2024. Exclusion criteria included MR examinations without DL datasets, spine imaging, and non-tumorous lesions (see Figure 1).

### 2.2. Imaging Protocol and Deep Learning Reconstruction Algorithm

All examinations were performed with a 1.5 T (MAGNETOM SOLA, Siemens, Siemens Healthineers, Erlangen, Germany) or a 3 T (MAGNETOM Vida Fit, Siemens Healthineers, Erlangen, Germany) clinical MRI scanner. Patients were scanned in the supine position with a 20-channel head coil on each scanner. The standard clinical protocol for postoperative imaging consists of FLAIR in the axial plane, T1-weighted imaging with and without contrast, T2-weighted imaging in the coronal plane, T2*-weighted imaging, diffusion-weighted imaging with two different b-values in the axial plane (0 s/mm^2^ and 1000 s/mm^2^), and corresponding apparent diffusion coefficient mapping. According to the standard clinical protocol, spin-echo sequences at 1.5 T were used for conventionally reconstructed T1-weighted sequences, while turbo spin-echo sequences (TSE) at 3 T were used for brain imaging. For the accelerated 1.5 T DL protocol, T1-weighted TSE sequences were used.

The undersampled DLR sequences were acquired and reconstructed separately for FLAIR, T1-weighted contrast-enhanced, and T2-weighted TSE imaging immediately after the corresponding conventional sequence type, with the same planes and slice thickness. The DLR algorithm Deep Resolve Boost by Siemens Healthineers (Erlangen, Germany) used in this study was cleared by the U.S. Food and Drug Administration (FDA) and has since been adopted in multiple investigations [1]. The software (XA50) offers a variational network approach, which alternates physics-based data consistency steps with learned convolutional neural network (CNN) regularization in a fixed, unrolled cascade. The CNN component, referred to as the “Deep, Iterative, Hierarchical Network,” extends a Down Up architecture through nested hierarchical blocks, allowing multi-resolution processing while conserving memory. A separate calibration acquisition supplies coil sensitivity maps and a bias field estimate to ensure homogeneous image intensity during reconstruction. Training relied on more than 25,000 fully sampled 2D slices obtained from volunteer examinations on a variety of clinical 1.5 T and 3 T MAGNETOM scanners (Siemens Healthineers, Erlangen, Germany). Once trained, the model was integrated into the scanner’s on-board reconstruction pipeline, enabling real-time assessment of its clinical utility. The Deep Resolve Boost algorithm only supports DLR for TSE readouts but not for SE. Hence, a SE sequence including DLR with improved acquisition time could not be acquired at 1.5 T.

For conventional reconstructed axial FLAIR (FLAIR_CR_), axial T1-weighted non-contrast-enhanced (T1_CR_) and contrast-enhanced (T1CE_CR_), and coronal T2-weighted images (T2_CR_), the acceleration factor was set to two phase encoding (PE) steps. In contrast, the acceleration factor was set to four PE steps for the DLR sequences: axial FLAIR (FLAIR_DLR_), axial T1-weighted non-contrast-enhanced (T1_DLR_) and contrast-enhanced (T1CE_DLR_), and coronal T2-weighted imaging (T2_DLR_). DLR was performed offline using the accessible standard clinical hardware infrastructure of the MRI workstation, with no discernible time impact on the clinical workflow. Table 1 and Table 2 provide a comprehensive overview of the acquisition parameters for these sequences and their DLR versions for 1.5 T and 3 T.

### 2.3. Qualitative Image Quality Analysis

The evaluation team consisted of four experienced raters: a neuroradiologist (Rater 1) with ten years of experience, a neurosurgeon (Rater 2) with sixteen years of experience, a neuro-oncologist (Rater 3) with eleven years of experience, and a radiation oncologist (Rater 4) with thirty years of experience. Before the qualitative evaluation of the patients included in this study, a training phase took place with five patients outside the study cohort to familiarize the raters with postoperative MRIs and the rating scores. The raters then independently assessed 33 image datasets, including CR and DLR images featuring axial FLAIR, coronal T2-weighted TSE, and axial T1-weighted imaging with and without contrast agent. The evaluations were conducted in a randomized order. Raters were blinded to the reconstruction type, clinical and radiological reports, and each other’s assessments, with all patient- or sequence-identifying markers eradicated to ensure anonymity. The study readings were performed in a certified reading room under controlled conditions on a dedicated workstation (GE Centricity PACS RA 1000, version 7.0.2; General Electric (GE) Healthcare, Chicago, IL, USA) and on certified diagnostic radiology monitors (RadiForce RX350, Eizo Corporation, Hakusan, Ishikawa, Japan). The four raters evaluated the datasets based on multiple parameters using a detailed Likert scale, ranging from 1 to 5, with 5 being the highest rating to ensure sufficient image quality gradation. The evaluation criteria for the 33 image datasets included image quality, tumor conspicuity (i.e., the delineation of the tumor), and diagnostic confidence. The following score was used to evaluate the overall image quality qualitatively: 1 = non-diagnostic images, 2 = poor image quality (significant blurring, noise, or artifacts that hinder interpretation, but some structures are still visible), 3 = fair image quality (noticeable blurring, noise, or artifacts that moderately affect interpretation), 4 = good image quality (minimal blurring, noise, or artifacts, allowing for effective interpretation), and 5 = excellent image quality (exceptionally clear with no blurring, noise, or artifacts, providing optimal conditions for interpretation). The visualization of the resection defect was rated with the following score: 1 = very poor conspicuity (the resection defect is almost indistinguishable from the surrounding tissue, making identification very difficult), 2 = poor conspicuity (the resection defect is faintly visible but blends significantly with the surrounding tissue, leading to uncertainty in identification), 3 = fair conspicuity (the resection defect is moderately visible but could be confused with surrounding structures, requiring careful analysis), 4 = good conspicuity (the resection defect is clearly visible and distinguishable from the surrounding tissue with minimal effort), and 5 = excellent conspicuity (the resection defect stands out prominently and is easily distinguishable from the surrounding tissue, ensuring confident identification). The degree of diagnostic confidence was evaluated with the following scheme: 1 = non-diagnostic images, 2 = low diagnostic confidence (confidence in the diagnosis is low, with significant doubt regarding findings and interpretation), 3 = moderate diagnostic confidence (there is moderate confidence in the diagnosis, with some uncertainty that requires further verification), 4 = high diagnostic confidence (there is high confidence in the diagnosis, with clear findings and minimal doubt), and 5 = excellent confidence (there is very high confidence in the diagnosis, with clear, definitive findings and no doubt). Each score incrementally reflected increasing levels of approval or quality, allowing for a nuanced assessment of the images. These assessments of both conventional and accelerated datasets were conducted in a random order across separate sessions, with at least a two-week washout period between each session to mitigate bias.

### 2.4. Rater Preference

In the preference analysis, each of the four experienced raters independently reviewed the image datasets two weeks after their initial analysis. The datasets encompassed FLAIR, T2-weighted TSE, non-enhanced, and contrast-enhanced T1-weighted sequences, which were evaluated on a per-patient basis. The raters were assigned to indicate their preferred sequence reconstruction type (CR vs. DLR) for individual sequence evaluations and an overall assessment of the entire dataset. The raters were unaware of the patients’ clinical information and the specific imaging techniques for each sequence.

### 2.5. Quantitative Image-Quality Indices

Image quality metrics (IQMs) were used to assess structural fidelity and noise sensitivity, utilizing publicly available formulas in MATLAB R2015b (The MathWorks, Inc., Natick, MA, USA). These include the Structural Similarity Index (SSIM) and the Multi-Scale SSIM (MS-SSIM), which were used to quantify the preservation of anatomical detail in DLR versus CR images as a reference. The Noise Quality Metric (NQM) provided a global measure of noise robustness, while the Feature Similarity Index (FSIM) captured the fidelity of high-frequency components, which are critical for delineating subtle postoperative changes. SNR and peak signal-to-noise ratio (PSNR) were also calculated [20]. The MATLAB source code for all IQM methods, except PSNR and SSIM, was obtained from the original authors [21,22,23,24]. We calculated PSNR and SSIM using the built-in functions of MATLAB.

### 2.6. Evaluation of Residual Tumor

The neuroradiologist, neurosurgeon, neuro-oncologist, and radiation oncologist independently reviewed each patient’s imaging datasets to assess residual tumor detection. These datasets consisted of FLAIR, T2-weighted TSE, and T1-weighted sequences (both non-enhanced and contrast-enhanced) with DLR or CR to determine the presence or absence of residual tumor compared to the preoperative images. Evaluations were performed on a per-patient basis.

### 2.7. Statistical Analysis

Statistical analysis was conducted using SPSS Statistics Version 30 (IBM, Armonk, NY, USA). Continuous variables were presented as mean ± standard deviation (SD), and ordinal variables as median and interquartile range (IQR). Image quality comparisons between groups were performed using the Wilcoxon signed-rank test. *p*-values from Wilcoxon signed-rank tests were reported unadjusted, in line with prior DLR image quality studies. Interrater reliability was evaluated using the Kendall coefficient of concordance (W). Kendall’s W is calculated by contrasting the number of concordant and discordant pairs, yielding a value between −1 and 1, where 0 indicates no correlation, 1 denotes perfect agreement, and −1 represents perfect disagreement. The effect sizes for Kendall’s W were categorized as follows: none (0 to <0.10), fair (0.10 to <0.30), moderate (0.30 to <0.50), large (0.50 to <0.70), and substantially large to almost perfect agreement (0.70–1.00). The significance level was set at alpha = 0.05.

## 3. Results

This retrospective study included 33 patients who underwent postoperative MRI after resection of an intracranial tumor, and all tumors were histopathologically confirmed. The mean age of the patients was 53.9 ± 20.3 years, including 14 males and 19 females. In total, 27% (9/25) of the patients had structural epilepsy due to the intracranial tumor. Comprehensive clinical data were available for all participants during the imaging study. Patient characteristics are shown in Table 3.

DLR sequences significantly reduced acquisition times compared to CR at 1.5 T and 3 T. At 1.5 T (Table 1), FLAIR, T2-weighted, T1-weighted, non-contrast, and contrast-enhanced T1-weighted sequences showed significant time savings ranging from 30% to 53%, with an overall mean reduction of 42%. At 3 T (Table 2), DLR sequences reduced scan times by approximately 27% to 31%, with an average decrease of 29%.

### 3.1. Image Quality-Based Analysis

**Figure 2:**
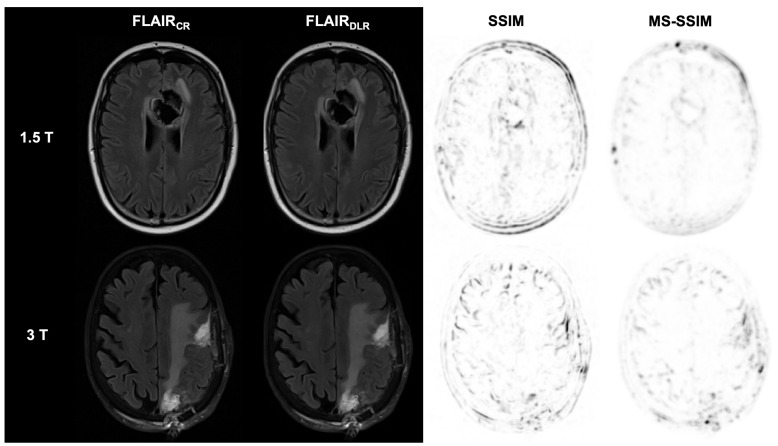
Postoperative imaging of a 55-year-old female patient with a glioblastoma (1.5 T) and an 80-year-old female patient with two histologically confirmed uterine carcinoma metastases in the left frontal and parietal lobes (3 T). The utilization of deep learning reconstruction (DLR) in conjunction with fluid-attenuated inversion recovery (FLAIR) images demonstrates non-inferior image quality and visualization of the resection defect, in addition to reduced image noise, compared to conventional reconstruction (CR). The Structural Similarity Index (SSIM) and Multi-Scale SSIM (MS-SSIM) are utilized to quantitatively assess the preservation of anatomical details in DLR images compared to CR images as reference images. CR: conventional reconstruction; DLR: deep learning reconstructed technique; FLAIR = fluid-attenuated inversion recovery images; and CE = contrast-enhanced.

**Figure 3:**
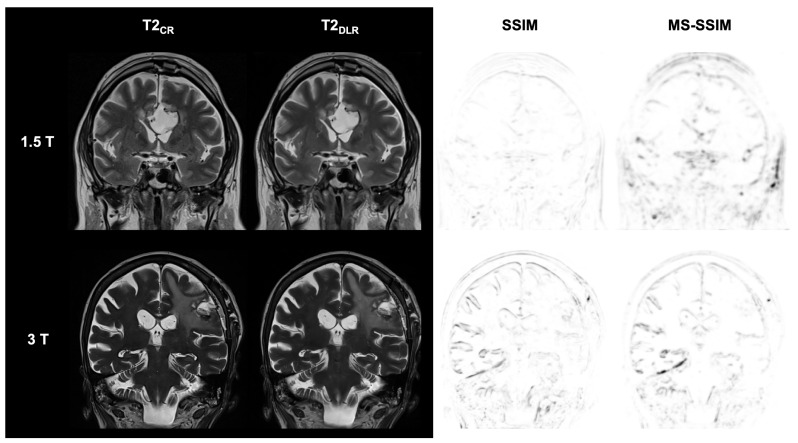
Postoperative imaging of a 55-year-old female patient with a glioblastoma (1.5 T) and an 80-year-old female patient with two histologically confirmed uterine carcinoma metastases in the left frontal and parietal lobes (3 T). The utilization of deep learning reconstruction (DLR) in conjunction with T2-weighted images demonstrates non-inferior image quality and visualization of the resection defect, in addition to reduced image noise, compared to conventional reconstruction (CR). Note the DLR algorithm’s tendency to produce images of the tumor’s internal structure that appear more uniform and softer than CR images. The Structural Similarity Index (SSIM) and Multi-Scale SSIM (MS-SSIM) are utilized to quantitatively assess the preservation of anatomical details in DLR images compared to CR images as reference images. CR: conventional reconstruction; DLR: deep learning reconstructed technique.

**Figure 4:**
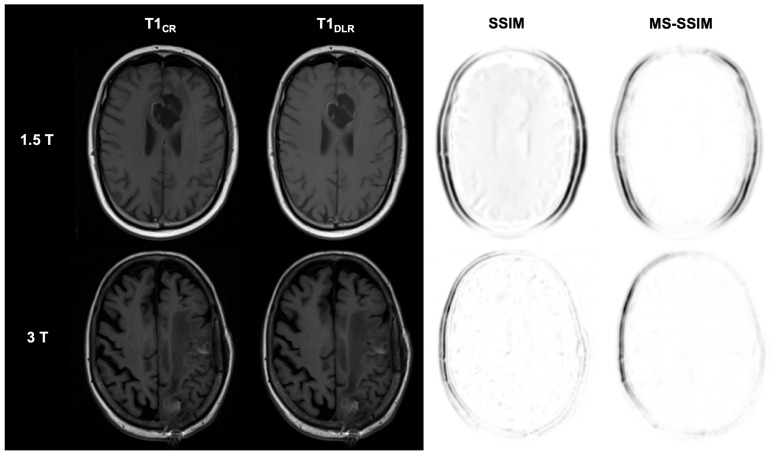
Postoperative imaging of a 55-year-old female patient with a glioblastoma (1.5 T) and an 80-year-old female patient with two histologically confirmed uterine carcinoma metastases in the left frontal and parietal lobes (3 T). The utilization of deep learning reconstruction (DLR) in conjunction with T1-weighted images demonstrates non-inferior image quality and visualization of the resection defect, in addition to reduced image noise, compared to conventional reconstruction (CR). According to the standard clinical protocol, spin-echo sequences are used at 1.5 T for the conventionally reconstructed sequences. In contrast, turbo spin-echo sequences are used for the DLR boost for technical reasons. The Structural Similarity Index (SSIM) and Multi-Scale SSIM (MS-SSIM) are utilized to quantitatively assess the preservation of anatomical details in DLR images compared to CR images as reference images. CR: conventional reconstruction; DLR: deep learning reconstructed technique; SSIM = Structural Similarity Index; and MS-SSIM = Multi-Scale SSIM.

**Figure 5:**
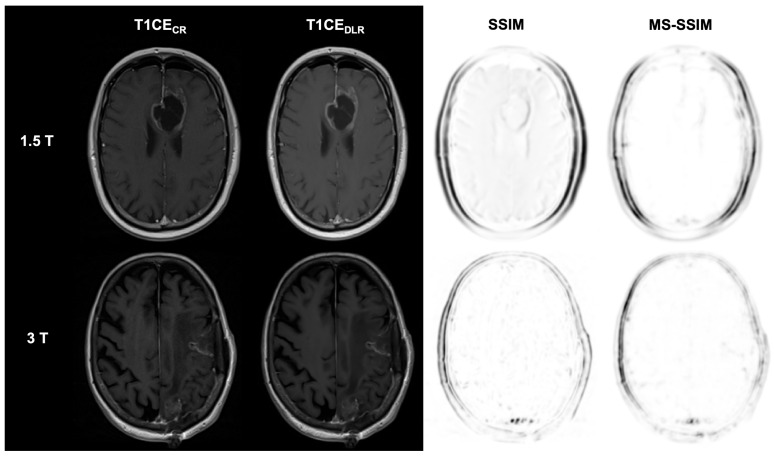
Postoperative imaging of a 55-year-old female patient with a glioblastoma (1.5 T) and an 80-year-old female patient with two histologically confirmed uterine carcinoma metastases in the left frontal and parietal lobes (3 T). The utilization of deep learning reconstruction (DLR) in conjunction with contrast-enhanced T1-weighted images (T1CE) demonstrates non-inferior image quality and visualization of the resection defect, as well as reduced image noise, compared to conventional reconstruction (CR). The Structural Similarity Index (SSIM) and Multi-Scale SSIM (MS-SSIM) are utilized to quantitatively assess the preservation of anatomical details in DLR images compared to CR images as reference images. CR: conventional reconstruction; DLR: deep learning reconstructed technique; CE = contrast-enhanced; SSIM = Structural Similarity Index; and MS-SSIM = Multi-Scale SSIM.

The analysis of qualitative image quality, visualization of the resection defect, and diagnostic confidence performed by the multidisciplinary team is presented in detail in Table 4, Table 5, Table 6 and Table 7 for FLAIR, T2-weighted, as well as T1-weighted and contrast-enhanced T1-weighted sequences. Examples of DLR and CR images from FLAIR, T2, non-contrast, and contrast-enhanced T1-weighted sequences at 1.5 T and 3 T are shown in Figure 2, Figure 3, Figure 4 and Figure 5.

For FLAIR imaging, overall image quality and visualization of the resection cavity improved significantly with DLR across all raters and both field strengths (*p* < 0.001), as shown in Table 4. In addition, diagnostic confidence was enhanced considerably (pooled *p* = 0.033 for 1.5 T, *p* = 0.002 for 3 T). However, rater-based analysis revealed discrepancies. Some raters reported only a tendency for significant changes in the visualization of the resection cavity, while others showed no significant changes in diagnostic accuracy, especially the neuroradiologist. Analysis of pooled data from 1.5 T and 3 T scans revealed significant ratings in all domains by all raters, except for the neuroradiologist and radiation oncologist, for diagnostic confidence (*p* = 0.157 and *p* = 0.480, respectively).

For T2-weighted imaging, overall image quality improved significantly with DLR for all raters at 1.5 T (*p* < 0.001 to *p* = 0.003) but not at 3 T (0.083 for the neurosurgeon to 0.565 for the neuroradiologist), as shown in Table 5. Visualization of the resection cavity was rated as superior for DLR by the neurosurgeon (*p* = 0.034) and the neuro-oncologist (*p* = 0.005). Diagnostic confidence did not change significantly for 1.5 T for all raters except the neuro-oncologist (*p* = 0.025). Visualization of the resection cavity and diagnostic confidence also did not change considerably for all raters at 3 T (*p* = 0.134 to 1).

For T1-weighted sequences, overall image quality was significantly improved with DLR at 1.5 T, except for the neurosurgeon, who rated the non-contrast-enhanced images similarly to CR (Table 6). Mixed results are shown for visualization of the resection cavity, and no significant changes are shown for diagnostic confidence. At 3 T, DLR has a more obvious tendency compared to CR.

For contrast-enhanced T1-weighted sequences, overall image quality, visualization of the resection cavity, and diagnostic confidence were rated as superior for DLR compared to CR at 1.5 T, except for the neurosurgeon, who rated image quality and diagnostic confidence as equal. At 3 T, overall image quality and diagnostic confidence were rated superior for DLR by all raters.

### 3.2. Pooled Analysis of Qualitative Image Quality Assessment

**Figure 6:**
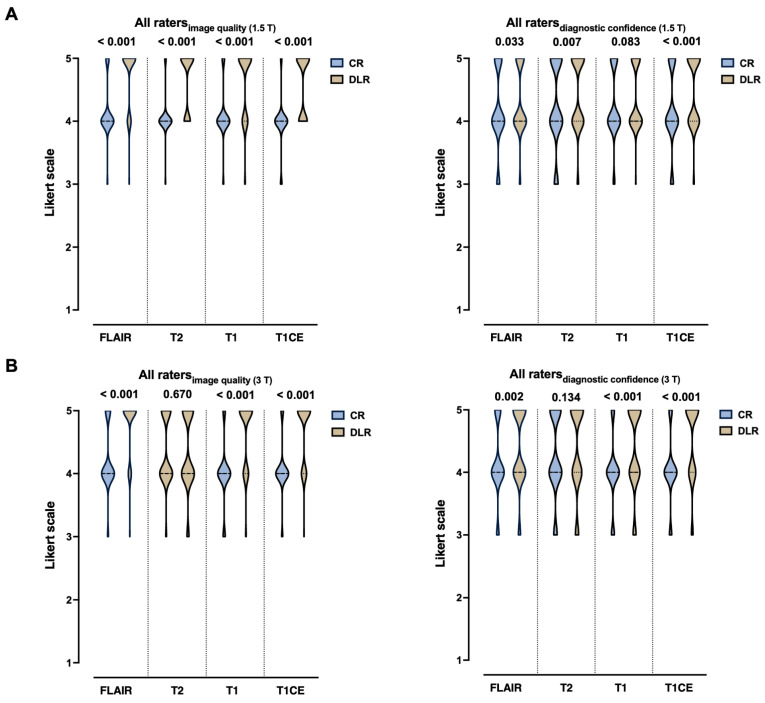
Multidisciplinary rating of overall image quality and diagnostic confidence of deep learning reconstructed images (DLR) and conventional recorded (CR) images by four experienced raters in a pooled data analysis for 1.5 T ((**A**), *n* = 17) and 3 T ((**B**), *n* = 16). Raters consist of a neuroradiologist (Rater 1), a neurosurgeon (Rater 2), a neuro-oncologist (Rater 3), and a radiation oncologist (Rater 4). Likert scale ranging from 1 to 5, with 5 being the best rating. Significant differences are indicated. The dotted lines represent the median. CR = conventional reconstruction; DLR = deep learning reconstructed technique; FLAIR = fluid-attenuated inversion recovery images; T2 = T2-weighted images; T1 = T1-weighted images. CE = contrast-enhanced; and T = Tesla.

Image quality was consistently rated higher for DLR imaging compared to CR imaging across all sequences by all raters in a pooled data analysis for 1.5 T (all *p* < 0.001), as shown in Figure 6A. Similar results are displayed for 3 T, except for T2-weighted images (*p* = 0.670). Regarding diagnostic confidence for 1.5 T, there was no significant difference between CR and DLR imaging for T1 (*p* = 0.083) but slightly significant differences for FLAIR (*p* = 0.033) and more significant changes for T2 (*p* = 0.007) and T1CE (*p* < 0.001) in favor of DLR, Figure 6A. Regarding diagnostic confidence for 3 T, there was a significant difference between CR and DLR imaging in favor of DLR for FLAIR (*p* = 0.002), T1, and T1CE (each *p* < 0.001). There were no significant differences for T2 (*p* = 0.134), as shown in Figure 6B. A more detailed insight into the results, including the mean (standard deviation) and median (interquartile ranges), can be found in Table 4, Table 5, Table 6 and Table 7 for each sequence.

### 3.3. Interrater Intraprotocol Agreement of Image Quality-Based Analysis

Interrater intraprotocol agreement among the four raters increased with DLR across most sequences and was more pronounced at 3 T than at 1.5 T, suggesting improved clarity and consistency in interpretation. Regarding overall image quality, at 1.5 T, Kendall’s W remained in the fair to moderate range. For 1.5 T, Kendall’s W for overall image quality_CR_ was 0.225 (0.131–0.331) vs. 0.231 (0.136–0.349) for the DLR protocol, visualization of the resection cavity_CR_ was 0.271 (0.150–0.362) vs. 0.280 (0.167–0.398), and diagnostic confidence_CR_ was 0.463 (0.368–0.560) vs. 0.361 (0.324- 0.469). At 3 T, agreement was higher with DLR, particularly for the visualization of the resection cavity (0.484–0.730 for DLR vs. 0.173–0.546 for CR. Kendall’s W for overall image quality_CR_ at 3 T increased from 0.276 (0.126–0.360) to 0.413 (0.135–0.605) for the DLR protocol, and diagnostic confidence_CR_ was 0.463 (0.193–0.518) for CR vs. 0.469 (0.394–0.576) for the DLR protocol. Table 8 shows the intraprotocol interrater agreement results for Kendall’s W for all protocols, including pooled data.

### 3.4. Rater Preference Between DLR and Conventionally Reconstructed Images

**Figure 7:**
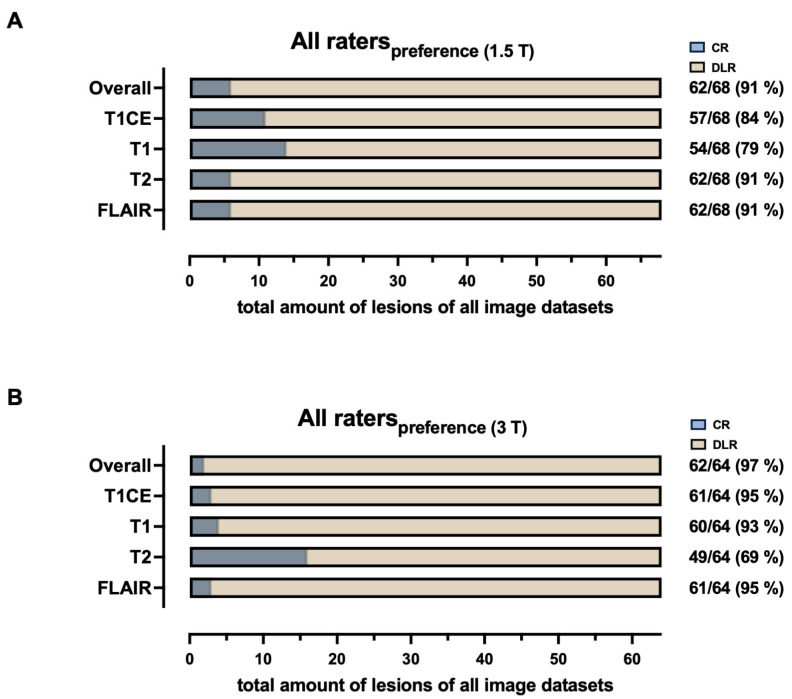
Pooled visual preferences in the review of paired magnetic resonance image sets following tumor resection at 1.5 T ((**A**), *n* = 17) and 3 T ((**B**), *n* = 16) by four experienced raters. Raters consist of a neuroradiologist (Rater 1), a neurosurgeon (Rater 2), a neuro-oncologist (Rater 3), and a radiation oncologist (Rater 4). CR = conventional reconstruction; DLR = deep learning reconstructed technique; FLAIR = fluid-attenuated inversion recovery images; T2 = T2-weighted images; T1 = T1-weighted images. CE = contrast-enhanced; and T = Tesla.

All four raters of the multidisciplinary team, comprising a neuroradiologist, a neurosurgeon, a neuro-oncologist, and a radiation oncologist (respectively, named rater 1 to 4), preferred DLR over CR images as a whole image set at 1.5 T and 3 T. The pooled data are shown in Figure 7. Individual ratings are shown in Supplementary Figure S1. Specifically, all raters (pooled) preferred FLAIR_DLR_ over FLAIR_CR_ (91% for 1.5 T, 95% for 3 T), T2_DLR_ over T2_CR_ (91% for 1.5 T, 69% for 3 T), T1_DLR_ over T1_CR_ (79% for 1.5 T, 93% for 3 T), and T1CE_DLR_ over T1CE_CR_ (79% for 1.5 T, 95% for 3 T). Overall preference for the DLR protocol was 91% for 1.5 T and 97% for 3 T.

### 3.5. Quantitative Image Quality Assessment

Detailed IQM results are documented in Table 9 for 1.5 T, 3 T, and pooled data. IQM results demonstrate high agreement for the entire image when CR is used as the reference and DLR as the comparison for SSIM and MS-SSIM at 1.5 T and 3 T. Regarding image quality metrics, both the Structural Similarity Index and Multi-Scale SSIM (on a 0–1 scale) were close to 1 for the entire image, indicating near-perfect similarity for both 1.5 and 3 T, despite the different sequence types for T1-weighted sequences at 1.5 T (SE for CR vs. TSE for DLR). The SSIM achieves a value of 0.9988 ± 0.0006 for FLAIR_pooled_, 0.9947 ± 0.0032 for T2_pooled_, 0.9882 ± 0.0047 for T1_pooled_, and 0.9838 ± 0.0060 for T1CE_pooled_, while the MS-SSIM achieves values of 0.9999 ± 0.0001, 0.9991 ± 0.0006, 0.9986 ± 0.0007, and 0.9980 ± 0.0009, respectively.

FSIM performs worse in terms of consistency between CR and DLR. It was found to be 0.8337 ± 0.0298 for FLAIR_pooled_, 0.8284 ± 0.0441 for T2_pooled_, 0.7584 ± 0.0555 for T1_pooled_, and 0.7427 ± 0.0509 for T1CE_pooled_. At 1.5 T, FSIM was 0.6860 ± 0.0763 for T1 and 0.6599 ± 0.0685 for T1CE.

Comparison of DLR versus CR sequences using NQM, SNR, and PSNR consistently showed higher values for DLR images. These findings align with the improved subjective image quality ratings, resection cavity delineation, and diagnostic confidence. However, as with FSIM, discrepancies in the T1-weighted sequences at 1.5 T to 3 T have also been observed. These discrepancies have resulted in reduced values for NQM, SNR, and PSNR in the comparison between CR and DLR. NQM was found to be 14.6983 ± 2.0582 for FLAIR_pooled_, 14.2008 ± 2.8076 for T2_pooled_, 9.5293 ± 2.5095 for T1_pooled_, and 9.1932 ± 2.1332 for T1CE_pooled_. SNR and PSNR achieved values of 14.2934 ± 2.1457 and 67.5261± 2.9485 for the whole image for FLAIR_pooled_, 13.4269 ± 2.1329 and 59.1513 ± 2.8639 for T2_pooled_, 10.0246 ± 1.7514 and 58.9975 ± 3.8395 for T1_pooled_, and 9.9720 ± 1.6580 and 56.6507 ± 3.4399 for T1CE_pooled_.

### 3.6. Evaluation of Residual Tumor

Table 10 demonstrates the results of the assessment of the detection rate between CR and DLR images of postoperative residual tumor. The analysis of residual tumor tissue revealed that CR does not detect more lesions than the DLR protocol.

The intrarater visual evaluation of residual tumor was comparable at 3 T between DLR and CR. In one case, the neurosurgeon (rater 2) evaluated residual postoperative tumor tissue in DLR images, whereas this lesion was not perceptible and was not rated in the CR series. For 1.5 T, residual tumor tissue was detected in DLR images and not in the corresponding CR sequences in no case for the neuroradiologist (rater 1), in four cases by the neurosurgeon (rater 2), in one case by the neuro-oncologist (rater 3), and in two cases by the radiation oncologist (rater 4). In all other cases, no residual tumor lesion was detected exclusively by either protocol.

## 4. Discussion

Although DLR is entering routine practice, real-world evidence remains limited. Because evaluations of therapeutic efficacy and related therapeutic decisions in neuro-oncology are multidisciplinary, the successful adoption of DLR depends on acceptance across specialties, not radiology alone. A review of the extant literature reveals that most studies on the qualitative and diagnostic performance of novel DL MRI have focused exclusively on radiologists. Integrating interdisciplinary research and collaboration between radiologists and other specialties can yield artificial techniques more closely aligned with clinical requirements.

Consequently, the present study quantitatively evaluated the differences between DLR and CR. In addition, four experienced raters from different specialties (i.e., neuroradiologist, neurosurgeon, neurooncologist, and radiotherapist) were asked to score qualitative assessments, such as image quality in postoperative MRIs, to evaluate specialty-dependent preferences and their effect on perceived residual tumor.

DL-based reconstruction methods have shown promise in decreasing the total scan time compared to traditional acceleration methods on standardized imaging setups by up to 60% [25,26]. DLR reduced scan time by 30–53% at 1.5 T and 27–31% at 3 T without compromising image quality or diagnostic confidence—findings that mirror earlier neuroradiological and non-neuroradiological assessments [7,10,27,28,29,30,31,32,33,34,35,36,37]. Across all specialties, DLR outperformed CR on both subjective and objective image quality metrics, underscoring its clinical utility in postoperative imaging.

This study offers several potential advantages, notably the evaluation conducted by a diverse team of medical professionals. This multidisciplinary approach ensures that the findings remain relevant and applicable across the diverse specialties involved in neuro-oncological imaging and clinical decision making. Surgical, medical, and radiation oncologists rely heavily on multiple imaging modalities for accurate diagnosis and effective cancer treatment planning and implementation [38]. Collaboration among cancer subspecialists is essential, as no single specialist can work effectively in isolation. Integrating services is crucial for optimizing treatment outcomes, minimizing complications, and enhancing long-term patient outcomes. Our data suggest that DLR can shorten scans, improve image quality, and maintain—or even increase—diagnostic confidence in neuro-oncological imaging protocols, consistent with prior work [7,10]. Combining quantitative metrics with qualitative evaluations provides a robust and comprehensive understanding of the imaging technique.

Lee et al. demonstrated that radiologists’ preference for DLR varies with experience, attributable in part to sharper edge rendition and unfamiliar image appearance, among other factors [37]. All raters in our cohort, across specialties and sequences, consistently preferred DLR over CR in most cases and judged its image quality to be good to excellent. Since neuroradiologists and other specialties rely on imaging daily for therapy decisions, the implications of this concordant preference are clinically meaningful. All raters agreed that DLR produced superior image quality with better delineated resection cavities, highlighted small enhancing foci, and improved contrast on T1-weighted images. There is a variation in preference for T1-weighted sequences at 1.5 T and T2-weighted sequences at 3 T. T2_DLR_ was preferred more often by all raters at 1.5 T than at 3 T, even though most still preferred DLR. Conversely, T1_CR_, both non- and contrast-enhanced, was preferred over T1_DLR_ in some cases at 1.5 T, whereas this was not the case at 3 T. The differences between the sequences are noteworthy and warrant further investigation. This discrepancy could be due to several factors. For T1-weighted images at 1.5 T, this may be explained by the different sequence types (SE vs. TSE) [39].

Furthermore, prior research has highlighted that DLR images exhibit lower noise levels than images obtained through traditional full-scan techniques. This trait can make DLR images unnatural to seasoned radiologists [40,41]. The DLR algorithm tends to produce images where the internal structure (e.g., of the tumor) appears more uniform and has a softer contrast than conventional images. Additionally, individual preferences and familiarity with the types of images commonly seen in regular practice also play a role in these differing evaluations. The qualitative image quality rating is supported by the image quality metrics SNR and PSNR, which are better for DLR than CR. PSNR expresses the log-scaled ratio of the maximum signal intensity to the reconstruction error. Higher PSNR, therefore, indicates reduced noise and sharper delineation. This is in line with other studies that have also shown improved SNR for DLR images [42,43,44,45].

Regarding image quality metrics, both SSIM and MS-SSIM were close to 1.0 (on a 0–1 scale) at both field strengths, indicating high similarity—even though 1.5 T T1-weighted scans used different sequence types (SE for CR vs. TSE for DLR). SSIM compares a test image (DLR) to a reference image (CR) by evaluating local luminance, contrast, and structural patterns. By evaluating multiple resolution levels and using an advanced pooling strategy, MS-SSIM further refines SSIM’s local measurements of image fidelity. The FSIM incorporates low-level features such as phase congruency and gradient magnitude and ranged from 0.824 to 0.848 at 3 T and from 0.660 to 0.842 at 1.5 T (on a 0–1 scale), with lower values for the 1.5 T T1-weighted images, likely reflecting sequence differences (SE for CR vs. TSE for DLR). This comparatively lower value underscores FSIM’s heightened sensitivity to subtle image differences. By typically enhancing high-frequency structure and edge sharpness, DLR boosts phase congruency. Thus, FSIM can vary markedly even when SSIM and MS-SSIM show only minor differences. The NQM index similarly accounts for luminance and contrast variations but also integrates the influence of spatial frequencies, viewing distance, and contrast masking. Similar effects for T1-weighted sequences at 1.5 T are also seen for NQM, with values of 3.1430 for contrast-enhanced T1-weighted sequences and 4.2984 for native T1-weighted sequences compared to 14.3721 for FLAIR and 14.1331 for T2-weighted TSE sequences. Although the NQM at 1.5 T is lower than FLAIR and the T2-weighted TSE sequence, a positive value still indicates improved image quality in DLR. Mason et al. reported that both FSIM and NQM showed consistently high correlations with radiologists’ subjective assessments of image quality, highlighting their effectiveness in capturing perceptual differences [20]. We could not verify this result in our study, which had considerably fewer datasets and only two comparison images.

The immediate postoperative MRI scan, obtained within 48 h of surgery, has been recommended by the European Association of Neuro-Oncology (EANO) and used, for example, as the baseline MRI in the Response Assessment in Neuro-Oncology (RANO) for newly diagnosed gliomas, including high-grade gliomas (RANO-HGG) and immunotherapy response assessment (iRANO) [17,46,47]. In contrast, the modified RANO (mRANO) criteria recommend using the first post-radiotherapy MRI as the baseline for newly diagnosed gliomas to minimize the effects of pseudoprogression and avoid challenges related to postoperative changes, corticosteroid variability, scan timing, and imaging techniques [48,49,50,51,52]. In support of this approach, Youssef et al. showed a greater correlation between progression-free survival and overall survival when the post-radiation scan was used as a baseline compared with the postoperative scan [53]. However, the difference was not statistically significant. If the patient is clinically stable, RANO 2.0 recommends a post-radiotherapy MRI around 4 weeks (21–35 days) after treatment as the baseline scan for newly diagnosed gliomas [54]. RANO 2.0 no longer treats the immediate postoperative scan as the response baseline; however, it remains valuable for detecting complications and gauging the extent of resection [55].

Accordingly, our findings demonstrate that an accelerated DLR protocol in the immediate postoperative setting is clinically relevant. Enhanced image quality may facilitate differentiation between actual residual tumor, postoperative change, and contrast leakage from the resection cavity. Evaluating how DLR influences individual assessments of residual tumor is essential for judging the comparability and interchangeability of reconstruction methods in routine practice. Importantly, no universally accepted imaging gold standard exists to define actual tumor extent or to determine whether a given reconstruction over- or underestimates it. Subjective comparisons between CR and DLR are crucial to ensure that DLR does not introduce spurious residual tumor findings. This study found that residual tumor was not detected in cases where DLR did not occur within the individual rater in the absence of CR alone. In the absence of these conditions, the detection of the residual tumor showed consistency among all raters, with DLR showing uniformity with CR images in the remaining cases at 3 T. This finding suggests that the residual tumor detected on DLR images was already visible on CR images, indicating that no additional findings were present. However, at both field strengths, there is variability in residual tumor detection between the disciplines. The reported non-detection of residual tumor is not the only issue; it may also reflect overinterpretation during evaluation, particularly by the neurosurgeon in four cases at 3 T (regardless of whether CR or DLR was used). At 1.5 T, visual assessment showed greater variability, with more residual tumor identified using DLR, especially by the neurosurgeon (*n* = 4). The neuro-oncologist identified one additional finding with DL images compared to the CR images, and the radiation oncologist identified two. This reflects the subjective nature of interpreting postoperative MRIs. In all other cases, DLR was comparable to CR in terms of the conspicuity of residual tumor. In summary, while DLR partially improved residual tumor perception for non-neuroradiologists, expert radiological interpretation—and hence downstream clinical decision making—was equivalent between DLR and CR.

The findings in this study should be interpreted with multiple limitations in mind. Firstly, this study included a relatively limited cohort of 33 patients from a single site, which was determined by power analysis. Despite this limitation, image quality and diagnostic confidence were significantly better for DLR among all patients. Additional patients from multiple sites would be needed to further validate the study’s generalizability. This is particularly evident and important in assessing residual tumor in individual disciplines at 1.5 T, presumably due to the better signal from the DLR, which might lead to a different assessment by those less experienced in imaging. The accelerated T1-weighted TSE sequence offers suboptimal gray-white contrast, but this is acceptable for the intended task of depicting enhancing lesions [39]. Secondly, an objective reference standard to confirm the presence of residual tumor is missing. Confirming residual tumor intraoperatively—particularly in low-grade gliomas—can be challenging, which is why some centers occasionally employ intraoperative MRI in selected brain-tumor cases. Furthermore, early post-therapy scans are deprioritized in mRANO and RANO 2.0 due to pseudoprogression and timing confounders. These sources would likely have added bias rather than resolved ground-truth uncertainty in this immediate postoperative cohort. Consequently, we concentrated on pre- versus immediate postoperative imaging to capture how different specialists perceive residual tumor—one of the primary aims of this study. Thirdly, focusing exclusively on postoperative MRIs may introduce selection bias, but it allowed us to isolate DLR performance in this clinically relevant cohort [11,56,57]. Furthermore, it is essential to highlight that CR images were set as the baseline and reference for the quantitative analysis. However, no universally accepted imaging standard definitively determines the actual size of tumors or residual tumor and which reconstruction technique potentially over- or underestimates residual tumor. Fourthly, qualitative assessments were conducted exclusively by a multidisciplinary team of experienced raters. This study did not include evaluations by inexperienced raters or comparisons between experienced and inexperienced raters, which could have provided additional insights. Qualitative evaluations are inherently susceptible to individual biases, which can potentially affect the reliability of the findings. We intentionally limited the present analysis to subspecialty-trained neuroradiologists and other senior clinicians to better isolate the technical performance of DLR from rater-experience effects in the context of postoperative MRIs. Additional specialists from these medical disciplines are therefore necessary to mitigate individual tendencies. They would further strengthen the findings of this study, potentially affect the conclusion, and help to better differentiate between the raters. Fifthly, despite blinding, experts could likely infer the reconstruction type, potentially introducing recognition bias. Sixthly, CR and DLR sequences were acquired sequentially, one immediately after the other, which is currently necessary when setting CR as the ground truth. However, this approach introduces a risk of non-identical image alignment, particularly in cases where patient motion occurs between acquisitions. We did not randomize the acquisition order of CR and DLR sequences to minimize potential motion- and alignment-related bias. In the current study, we did not observe any relevant motion artifacts. Lastly, this study was limited to one manufacturer’s MRI scanners, which restricted the generalizability of the results to other settings. “True” reproducibility—i.e., across platforms from different manufacturers—cannot be realized within a single-centered setting.

## 5. Conclusions

An accelerated deep learning reconstruction (DLR) protocol for postoperative brain MRI proved clinically feasible, cutting acquisition time by 42% at 1.5 T and 29.6% at 3 T. Across all sequences (FLAIR, T2, T1, T1CE), DLR achieved higher qualitative and quantitative image quality scores than conventional reconstruction, and at 1.5 T it occasionally altered individual raters’ perception of residual tumor. Based on the results of this study, we believe DL-based reconstruction in postoperative MRI could lead to easier-to-interpret images in some cases. Validation in larger, dedicated postoperative cohorts is still required. Furthermore, our multidisciplinary approach indicates that not only neuroradiologists but also neurosurgeons, neuro-oncologists, and radiotherapists involved in treating and assessing treatment response preferred DLR over CR images. The strong preference for DLR sequences among all raters highlights the potential for these advanced imaging techniques to enhance diagnostic and therapeutic decision making in neuro-oncological practice and promote the adoption of this approach for implementation in clinical routine.

## Figures and Tables

**Table 1:** MRI acquisition parameters at 1.5 Tesla.

Parameters	FLAIR_CR_	FLAIR_DLR_	T2(TSE)_CR_	T2(TSE)_DLR_	T1(SE)_CR_	T1(TSE)_DLR_	T1(SE)CE_CR_	T1(TSE)CE_DLR_
Field of view (mm)	230	230	170	170	230	230	230	230
Voxel size (mm)	0.4 × 0.4 × 4.0	0.4 × 0.4 × 4.0	0.3 × 0.3 × 3.0	0.3 × 0.3 × 3.0	0.4 × 0.4 × 4.0	0.4 × 0.4 × 4.0	0.4 × 0.4 × 4.0	0.4 × 0.4 × 4.0
Slice thickness (mm)	4	4	4	4	4	4	4	4
Number of slices	36	36	34	34	36	36	36	36
Base resolution	256	256	256	256	256	256	256	256
Parallel imaging factor	n.a.	4	n.a.	4	n.a.	3	n.a.	3
Acceleration mode	None	GRAPPA	None	GRAPPA	None	GRAPPA	None	GRAPPA
TR (ms)	8800	8800	3550	3550	450	600	450	600
TE (ms)	118	118	104	104	12	9.6	12	9.6
Averages	1	1	2	2	1	2	1	2
Concatenations	2	2	2	2	2	3	2	3
Acquisition time (min)	2:58	1:23	2:45	1:23	2:28	1:43	2:28	1:43
Time savings using DLR sequences in min (%)		1:35 (53%)		1:22 (50%)		0:45 (30%)		0:45 (30%)
Time saving on average in min (%)				1:07 (42%)				
Total time saving in min				4:27				

FLAIR = fluid-attenuated inversion recovery; CR = conventional reconstruction; DLR = deep learning reconstructed technique; CE = contrast-enhanced; GRAPPA = GeneRalized Autocalibrating Partial Parallel Acquisition (parallel imaging technique); TR = repetition time; TE = echo time; SE = spin echo; TSE = turbo spin echo; mm = millimeter; ms = millisecond; n.a. = not applicable; and min = minute.

**Table 2:** MRI acquisition parameters at 3 Tesla.

Parameters	FLAIR_CR_	FLAIR_DLR_	T2(TSE)_CR_	T2(TSE)_DLR_	T1(TSE)_CR_	T1(TSE)_DLR_	T1(TSE)CE_CR_	T1(TSE)CE_DLR_
Field of view (mm)	230	230	230	230	230	230	230	230
Voxel size (mm)	0.4 × 0.4 × 4.0	0.4 × 0.4 × 4.0	0.2 × 0.2 × 3.0	0.2 × 0.2 × 3.0	0.4 × 0.4 × 4.0	0.4 × 0.4 × 4.0	0.4 × 0.4 × 4.0	0.4 × 0.4 × 4.0
Slice thickness (mm)	4	4	4	4	4	4	4	4
Number of slices	36	36	34	34	36	36	36	36
Base resolution	320	320	384	384	304	304	304	304
Parallel imaging factor	2	4	2	4	2	4	2	4
Acceleration mode	GRAPPA	GRAPPA	GRAPPA	GRAPPA	GRAPPA	GRAPPA	GRAPPA	GRAPPA
TR (ms)	8800	8800	7640	7640	2000	2000	2000	2000
TE (ms)	81	81	87	87	9.7	9.7	9.7	9.7
Averages	1	1	2	2	1	1	1	1
Concatenations	2	2	1	1	2	2	2	2
Acquisition time (min)	2:40	1:57	2:11	1:36	1:54	1:19	1:54	1:19
Time savings using DLR sequences in min (%)		0:43 (27%)		0:35 (27%)		0:35 (31%)		0:35 (31%)
Time saving on average in min (%)				0:37 (29%)				
Total time saving in min				2:28				

FLAIR = fluid-attenuated inversion recovery; CR = conventional reconstruction; DLR = deep learning reconstructed technique; CE = contrast-enhanced; GRAPPA = GeneRalized Autocalibrating Partial Parallel Acquisition (parallel imaging technique); TR = repetition time; TE = echo time; TSE = turbo spin echo; mm = millimeter; ms = millisecond; and min = minute.

**Table 3:** Patient’s characteristics.

Characteristics	Values
Number of examinations	*n* = 33
1.5 T	*n* = 17
3 T	*n* = 16
Age, mean ± standard deviation (years)	53.9 ± 20.3
1.5 T	56.4 ± 21.7
3 T	51.1 ± 19.6
Sex (male vs. female)	*n* = 14 (42%) vs. *n* = 19 (58%)
1.5 T	*n* = 8 (47%) vs. *n* = 9 (53%)
3 T	*n* = 6 (37%) vs. *n* = 10 (63%)
Structural epilepsy	*n* = 9/33 (27%)
1.5 T	*n* = 3/17 (18%)
3 T	*n* = 6/16 (38%)
Tumor type (lesions), 1.5 T	
Metastasis	6
Subependymoma	1
DNET	1
Pilocytic astrocytoma	1
Astrocytoma grade 2	1
Astrocytoma grade 4	1
Oligodendroglioma grade 3	2
Glioblastoma	4
Tumor type (lesions), 3 T	
Metastasis	5
Ependymoma	1
Hemangiopericytoma	1
Ganglioglioma grade 1	1
Midline glioma grade 4	1
Astrocytoma grade 2	1
Glioblastoma	6
Previous surgeries	*n* = 10/33 (30%)
1.5 T	*n* = 4/17 (24%)
3 T	*n* = 6/16 (38%)
Contrast-enhancing tumor parts	*n* = 27/33 (82%)
1.5 T	*n* = 12/17 (71%)
3 T	*n* = 15/16 (94%)
Karnofsky performance scale index (KPS in %), median [range]	80 [20–100]
1.5 T	80 [40–90]
3 T	80 [20–100]
ECOG performance status scale, median [range]	1 [0–4]
1.5 T	1 [0–4]
3 T	1 [0–4]

ECOG = Eastern Cooperative Oncology Group; T = Tesla.

**Table 4:** Multidisciplinary qualitative rating of the overall image quality, the visualization of the resection cavity, and diagnostic confidence of conventional and deep learning reconstructed FLAIR images at 1.5 Tesla (T, *n* = 17) and 3 T (*n* = 16) by four experienced raters. Raters consist of a neuroradiologist (Rater 1), a neurosurgeon (Rater 2), a neuro-oncologist (Rater 3), and a radiation oncologist (Rater 4). The *p*-values are calculated using the Wilcoxon signed-rank test.

		1.5 T (*n* = 17)					3 T (*n* = 16)					Pooled (*n* = 33)				
		FLAIR_CR_		FLAIR_DLR_			FLAIR_CR_		FLAIR_DLR_			FLAIR_CR_		FLAIR_DLR_		
		Mdn (IQR)	M ± SD	Mdn (IQR)	M ± SD	*p*-Value FLAIR_CR_ vs. FLAIR_DLR_	Mdn (IQR)	M ± SD	Mdn (IQR)	M ± SD	*p*-Value FLAIR_CR_ vs. FLAIR_DLR_	Mdn (IQR)	M ± SD	Mdn (IQR)	M ± SD	*p*-Value FLAIR_CR_ vs. FLAIR_DLR_
Overall image quality	Rater 1	4 (4–5)	4.24 ± 0.437	5 (4.5–5)	4.76 ± 0.437	0.003	4 (4–5)	4.31 ± 0.602	5 (5–5)	4.88 ± 0.342	0.007	4 (4–5)	4.27 ± 0.517	5 (5–5)	4.82 ± 0.392	<0.001
	Rater 2	4 (4–4.5)	4.24 ± 0.437	5 (5–5)	4.94 ± 0.243	<0.001	4 (4–5)	4.31 ± 0.479	5 (5–5)	4.94 ± 0.250	0.002	4 (4–5)	4.27 ± 0.452	5 (5–5)	4.94 ± 0.242	<0.001
	Rater 3	4 (4–4)	4.00 ± 0.500	5 (4–5)	4.53 ± 0.624	0.013	4 (4–4)	4.00 ± 0.365	5 (4–5)	4.56 ± 0.629	0.007	4 (4–4)	4.00 ± 0.433	5 (4–5)	4.55 ± 0.617	<0.001
	Rater 4	4 (4–4)	4.18 ± 0.393	5 (4–5)	4.76 ± 0.437	0.002	4 (4–5)	4.25 ± 0.577	5 (4.25–5)	4.75 ± 0.447	0.011	4 (4–4.5)	4.21 ± 0.485	5 (4.5–5)	4.76 ± 0.435	<0.001
	All Raters	4 (4–4)	4.16 ± 0.444	5 (5–5)	4.75 ± 0.469	<0.001	4 (4–5)	4.22 ± 0.519	5 (5–5)	4.75 ± 0.453	<0.001	4 (4–4)	4.19 ± 0.481	5 (5–5)	4.77 ± 0.460	<0.001
Visualization of the resection cavity	Rater 1	4 (4–5)	4.24 ± 0.562	5 (4–5)	4.53 ± 0.514	0.059	4 (4–5)	4.25 ± 0.683	4 (4–5)	4.38 ± 0.500	0.317	4 (4–5)	4.24 ± 0.614	5 (4–5)	4.45 ± 0.506	0.035
	Rater 2	4 (4–5)	4.29 ± 0.470	5 (4–5)	4.65 ± 0.493	0.034	4.5 (4–5)	4.44 ± 0.629	5 (5–5)	4.88 ± 0.342	0.088	4 (4–5)	4.36 ± 0.549	5 (4–4)	4.76 ± 0.435	<0.001
	Rater 3	4 (3.5–4)	3.82 ± 0.529	4 (4–4.5)	4.12 ± 0.600	0.059	4 (3–4)	3.62 ± 0.500	4 (4–4)	3.94 ± 0.443	0.059	4 (3–4)	3.73 ± 0.517	4 (4–4)	4.03 ± 0.529	0.008
	Rater 4	4 (4–4.5)	4.18 ± 0.529	4 (4–5)	4.47 ± 0.514	0.025	4 (3.25–5)	4.13 ± 0.806	4 (4–5)	4.31 ± 0.602	0.180	4 (4–5)	4.15 ± 0.667	4 (4–4)	4.39 ± 0.556	0.011
	All Raters	4 (4–4)	4.13 ± 0.544	4 (4–5)	4.44 ± 0.557	<0.001	4 (4–5)	4.11 ± 0.715	4 (4–5)	4.38 ± 0.577	<0.001	4 (4–5)	4.12 ± 0.630	4 (4–5)	4.41 ± 0.556	<0.001
Diagnostic confidence	Rater 1	4 (4–5)	4.47 ± 0.514	5 (4–5)	4.53 ± 0.514	0.564	4 (4–5)	4.31 ± 0.479	4.5 (4–5)	4.50 ± 0.516	0.180	4 (4–5)	4.39 ± 0.496	5 (4–5)	4.52 ± 0.508	0.157
	Rater 2	4 (4–5)	4.29 ± 0.470	4 (4–5)	4.47 ± 0.514	0.083	5 (4–5)	4.50 ± 0.632	5 (5–5)	4.88 ± 0.342	0.014	4 (4–5)	4.39 ± 0.556	5 (4–5)	4.67 ± 0.479	0.003
	Rater 3	4 (3–4)	3.59 ± 0.618	4 (4–4)	3.86 ± 0.485	0.025	4 (3–4)	3.62 ± 0.500	4 (3–4)	3.75 ± 0.577	0.317	4 (3–4)	3.61 ± 0.566	4 (3.5–4)	3.82 ± 0.528	0.020
	Rater 4	4 (4–5)	4.47 ± 0.514	4 (4–5)	4.41 ± 0.507	0.564	4 (4–4.75)	4.25 ± 0.447	4 (4–5)	4.44 ± 0.512	0.180	4 (4–5)	4.36 ± 0.489	4 (4–5)	4.42 ± 0.502	0.480
	All Raters	4 (4–5)	4.21 ± 0.636	4 (4–5)	4.32 ± 0.558	0.033	4 (4–5)	4.17 ± 0.606	4 (4–5)	4.39 ± 0.663	0.002	4 (4–5)	4.19 ± 0.619	4 (4–5)	4.36 ± 0.594	<0.001

CR = conventional reconstruction; DLR = deep learning reconstruction; Mdn = median; IQR = interquartile range; M = mean; SD = standard deviation; FLAIR = fluid-attenuated inversion recovery; and T = Tesla.

**Table 5:** Multidisciplinary qualitative rating of the overall image quality, the visualization of the resection cavity, and diagnostic confidence of conventional and deep learning reconstructed T2-weighted images at 1.5 Tesla (T, *n* = 17) and 3 T (*n* = 16) by four experienced raters. Raters consist of a neuroradiologist (Rater 1), a neurosurgeon (Rater 2), a neuro-oncologist (Rater 3), and a radiation oncologist (Rater 4). The *p*-values are calculated using the Wilcoxon signed-rank test.

		1.5 T (*n* = 17)					3 T (*n* = 16)					Pooled (*n* = 33)				
		T2_CR_		T2_DLR_			T2_CR_		T2_DLR_			T2_CR_		T2_DLR_		
		Mdn (IQR)	M ± SD	Mdn (IQR)	M ± SD	*p*-Value T2_CR_ vs. T2_DLR_	Mdn (IQR)	M ± SD	Mdn (IQR)	M ± SD	*p*-Value T2_CR_ vs. T2_DLR_	Mdn (IQR)	M ± SD	Mdn (IQR)	M ± SD	*p*-Value T2_CR_ vs. T2_DLR_
Overall image quality	Rater 1	4 (4–4)	4.12 ± 0.332	5 (4–5)	4.65 ± 0.493	0.003	4 (4–5)	4.38 ± 0.500	4 (4–5)	4.31 ± 0.479	0.565	4 (4–4.5)	4.24 ± 0.435	4 (4–5)	4.48 ± 0.508	0.021
	Rater 2	4 (4–4)	4.18 ± 0.393	5 (5–5)	4.94 ± 0.243	<0.001	5 (4.25–5)	4.75 ± 0.447	5 (5–5)	4.94 ± 0.250	0.083	4 (4–5)	4.45 ± 0.506	5 (5–5)	4.94 ± 0.242	<0.001
	Rater 3	4 (4–4)	3.94 ± 0.243	5 (4–5)	4.53 ± 0.514	0.002	4 (4–4)	4.06 ± 0.574	4 (4–4)	3.94 ± 0.574	0.527	4 (4–4)	4.00 ± 0.433	4 (4–5)	4.24 ± 0.614	0.074
	Rater 4	4 (4–4)	4.00 ± 0.354	5 (4–5)	4.59 ± 0.507	0.002	4 (4–5)	4.31 ± 0.479	4 (4–5)	4.19 ± 0.655	0.414	4 (4–4)	4.15 ± 0.442	4 (4–5)	4.39 ± 0.609	0.046
	All Raters	4 (4–4)	4.06 ± 0.340	5 (4–5)	4.68 ± 0.471	<0.001	4 (4–5)	4.37 ± 0.549	4 (4–5)	4.34 ± 0.623	0.670	4 (4–4)	4.21 ± 0.479	5 (4–5)	4.52 ± 0.573	<0.001
Visualization of the resection cavity	Rater 1	4 (4–5)	4.35 ± 0.493	5 (4–5)	4.59 ± 0.507	0.102	4 (4–4.75)	4.13 ± 0.619	4 (4–4.75)	4.19 ± 0.544	0.564	4 (4–5)	4.24 ± 0.561	4 (4–5)	4.39 ± 0.556	0.096
	Rater 2	4 (4–5)	4.35 ± 0.606	5 (4.5–5)	4.71 ± 0.588	0.034	5 (5–5)	4.81 ± 0.403	5 (5–5)	4.94 ± 0.250	0.157	5 (4–5)	4.58 ± 0.561	5 (5–5)	4.82 ± 0.465	0.011
	Rater 3	4 (3.5–4)	3.76 ± 0.437	4 (4–5)	4.24 ± 0.562	0.005	4 (3–4)	3.75 ± 0.683	4 (4–4)	3.87 ± 0.500	0.414	4 (3–4)	3.76 ± 0.561	4 (4–4)	4.06 ± 0.556	0.008
	Rater 4	4 (4–5)	4.24 ± 0.562	5 (4–5)	4.53 ± 0.514	0.059	4 (4–5)	4.25 ± 0.683	4 (4–4.75)	4.19 ± 0.544	0.564	4 (4–5)	4.24 ± 0.614	4 (4–5)	4.36 ± 0.549	0.206
	All Raters	4 (4–5)	4.18 ± 0.571	5 (4–5)	4.51 ± 0.560	<0.001	4 (4–5)	4.23 ± 0.707	4 (4–5)	4.30 ± 0.609	0.285	4 (4–5)	4.20 ± 0.639	4 (4–5)	4.41 ± 0.592	<0.001
Diagnostic confidence	Rater 1	5 (4–5)	4.53 ± 0.624	5 (4–5)	4.65 ± 0.493	0.414	4 (4–5)	4.37 ± 0.619	4.5 (4–5)	4.38 ± 0.719	1	5 (4–5)	4.45 ± 0.617	5 (4–5)	4.52 ± 0.619	0.527
	Rater 2	4 (4–5)	4.35 ± 0.606	5 (4–5)	4.53 ± 0.624	0.083	5 (5–5)	4.81 ± 0.403	5 (5–5)	4.94 ± 0.250	0.157	5 (4–5)	4.58 ± 0.561	5 (4.5–5)	4.73 ± 0.517	0.025
	Rater 3	4 (3–4)	3.71 ± 0.588	4 (4–4)	4.00 ± 0.500	0.025	4 (3–4)	3.69 ± 0.602	4 (3.25–4)	3.88 ± 0.619	0.180	4 (3–4)	3.70 ± 0.585	4 (4–4)	3.94 ± 0.556	0.011
	Rater 4	5 (4–5)	4.53 ± 0.624	5 (4–5)	4.65 ± 0.493	0.414	4 (4–5)	4.25 ± 0.577	4 (4–5)	4.31 ± 0.704	0.655	4 (4–5)	4.39 ± 0.609	5 (4–5)	4.48 ± 0.619	0.366
	All Raters	4 (4–5)	4.28 ± 0.688	4.5 (4–5)	4.46 ± 0.584	0.007	4 (4–5)	4.28 ± 0.678	4.5 (4–5)	4.38 ± 0.701	0.134	4 (4–5)	4.28 ± 0.680	4.5 (4–5)	4.42 ± 0.643	0.003

CR = conventional reconstruction; DLR = deep learning reconstruction; Mdn = median; IQR = interquartile range; M = mean; SD = standard deviation; T2 = T2-weighted images; and T = Tesla.

**Table 6:** Multidisciplinary qualitative rating of the overall image quality, the visualization of the resection cavity, and diagnostic confidence of conventional and deep learning reconstructed T1-weighted images at 1.5 Tesla (T, *n* = 17) and 3 T (*n* = 16) by four experienced raters. Raters consist of a neuroradiologist (Rater 1), a neurosurgeon (Rater 2), a neuro-oncologist (Rater 3), and a radiation oncologist (Rater 4). The *p*-values are calculated using the Wilcoxon signed-rank test.

		1.5 T (*n* = 17)					3 T (*n* = 16)					Pooled (*n* = 33)				
		T1_CR_		T1_DLR_			T1_CR_		T1_DLR_			T1_CR_		T1_DLR_		
		Mdn (IQR)	M ± SD	Mdn (IQR)	M ± SD	*p*-Value T1_CR_ vs. T1_DLR_	Mdn (IQR)	M ± SD	Mdn (IQR)	M ± SD	*p*-Value T1_CR_ vs. T1_DLR_	Mdn (IQR)	M ± SD	Mdn (IQR)	M ± SD	*p*-Value T1_CR_ vs. T1_DLR_
Overall image quality	Rater 1	4 (4–4)	4.06 ± 0.243	5 (4–5)	4.71 ± 0.470	0.002	4 (4–4)	4.13 ± 0.342	5 (5–5)	4.81 ± 0.403	<0.001	4 (4–4)	4.09 ± 0.292	5 (4.5–5)	4.76 ± 0.435	<0.001
	Rater 2	4 (4–5)	4.41 ± 0.506	5 (4–5)	4.65 ± 0.493	0.317	4 (4–4.75)	4.25 ± 0.447	5 (5–5)	5.00	<0.001	4 (4–5)	4.33 ± 0.479	5 (5–5)	4.82 ± 0.392	0.002
	Rater 3	4 (4–4)	3.88 ± 0.485	5 (4–5)	4.41 ± 0.712	0.020	4 (3–4)	3.56 ± 0.512	4 (4–4)	4.06 ± 0.574	0.021	4 (3–4)	3.73 ± 0.517	4 (4–5)	4.24 ± 0.663	0.001
	Rater 4	4 (4–4)	4.06 ± 0.243	5 (5–5)	4.82 ± 0.363	<0.001	4 (4–4)	4.06 ± 0.443	5 (4.25–5)	4.75 ± 0.447	0.002	4 (4–4)	4.06 ± 0.348	5 (5–5)	4.79 ± 0.425	<0.001
	All Raters	4 (4–4)	4.10 ± 0.428	5 (4–5)	4.65 ± 0.540	<0.001	4 (4–4)	4.00 ± 0.504	5 (4–5)	4.66 ± 0.541	<0.001	4 (4–4)	4.05 ± 0.467	5 (4–5)	4.65 ± 0.538	<0.001
Visualization of the resection cavity	Rater 1	4 (4–4)	4.00 ± 0.612	5 (4–5)	4.65 ± 0.493	0.002	4 (3–4.75)	3.81 ± 0.834	4 (3.25–5)	4.13 ± 0.806	0.025	4 (3–4)	3.91 ± 0.723	5 (4–5)	4.39 ± 0.704	<0.001
	Rater 2	4 (4–5)	4.41 ± 0.507	4 (4–5)	4.47 ± 0.514	0.665	4 (4–4.75)	4.19 ± 0.544	5 (5–5)	4.88 ± 0.342	<0.001	4 (4–5)	4.30 ± 0.529	5 (4–5)	4.67 ± 0.479	0.003
	Rater 3	4 (4–4)	3.88 ± 0.485	4 (4–4.5)	4.12 ± 0.600	0.102	3.5 (3–4)	3.50 ± 0.516	4 (3–4)	3.81 ± 0.655	0.059	4 (3–4)	3.70 ± 0.529	4 (4–4)	3.97 ± 0.637	0.013
	Rater 4	4 (4–4)	4.00 ± 0.612	5 (4–5)	4.59 ± 0.507	<0.001	3.5 (3–4.75)	3.75 ± 0.856	4 (3–5)	4.00 ± 0.894	0.046	4 (3–4)	3.88 ± 0.740	4 (4–5)	4.30 ± 0.770	<0.001
	All Raters	4 (4–4)	4.07 ± 0.581	4 (4–5)	4.46 ± 0.558	<0.001	4 (3–4)	3.81 ± 0.732	4 (4–5)	4.20 ± 0.800	<0.001	4 (3.25–4)	3.95 ± 0.669	4 (4–5)	4.33 ± 0.695	<0.001
Diagnostic confidence	Rater 1	4 (4–5)	4.41 ± 0.507	4 (4–5)	4.47 ± 0.514	0.317	4 (4–4.75)	4.19 ± 0.544	4 (4–5)	4.38 ± 0.500	0.083	4 (4–5)	4.30 ± 0.529	4 (4–5)	4.42 ± 0.502	0.046
	Rater 2	4 (4–5)	4.41 ± 0.507	4 (4–5)	4.35 ± 0.493	0.564	4 (4–4.75)	4.19 ± 0.544	5 (5–5)	4.88 ± 0.432	<0.001	4 (4–5)	4.30 ± 0.529	5 (4–5)	4.61 ± 0.496	0.008
	Rater 3	4 (3–4)	3.76 ± 0.562	4 (4–4)	4.06 ± 0.556	0.059	4 (3–4)	3.69 ± 0.479	4 (3–4)	3.69 ± 0.602	1	4 (3–4)	3.73 ± 0.517	4 (3.5–4)	3.88 ± 0.600	0.096
	Rater 4	4 (4–5)	4.41 ± 0.507	4 (4–5)	4.47 ± 0.514	0.317	4 (4–4)	3.94 ± 0.574	5 (4–5)	4.56 ± 0.629	0.002	4 (4–5)	4.18 ± 0.584	5 (4–5)	4.52 ± 0.566	<0.001
	All Raters	4 (4–5)	4.25 ± 0.583	4 (4–5)	4.34 ± 0.536	0.083	4 (4–4)	4.00 ± 0.563	4 (4–5)	4.37 ± 0.678	<0.001	4 (4–4)	4.13 ± 0.585	4 (4–5)	4.36 ± 0.607	<0.001

CR = conventional reconstruction; DLR = deep learning reconstruction; Mdn = median; IQR = interquartile range; M = mean; SD = standard deviation; T1 = T1-weighted images; and T = Tesla.

**Table 7:** Multidisciplinary qualitative rating of the overall image quality, visualization of the resection cavity, and diagnostic confidence of conventional and deep learning reconstructed contrast-enhanced T1-weighted images at 1.5 Tesla (T, *n* = 17) and 3 T (*n* = 16) by four experienced raters. Raters consist of a neuroradiologist (Rater 1), a neurosurgeon (Rater 2), a neuro-oncologist (Rater 3), and a radiation oncologist (Rater 4). The *p*-values were calculated using the Wilcoxon signed-rank test.

		1.5 T (*n* = 17)					3 T (*n* = 16)					Pooled (*n*= 33)				
		T1CE_CR_		T1CE_DLR_			T1CE_CR_		T1CE_DLR_			T1CE_CR_		T1CE_DLR_		
		Mdn (IQR)	M ± SD	Mdn (IQR)	M ± SD	*p*-Value T1CE_CR_ vs. T1CE_DLR_	Mdn (IQR)	M ± SD	Mdn (IQR)	M ± SD	*p*-Value T1CE_CR_ vs. T1CE_DLR_	Mdn (IQR)	M ± SD	Mdn (IQR)	M ± SD	*p*-Value T1CE_CR_ vs. T1CE_DLR_
Overall image quality	Rater 1	4 (4–4)	4.06 ± 0.243	5 (5–5)	4.82 ± 0.393	<0.001	4 (4–4)	4.19 ± 0.403	5 (4.25–5)	4.75 ± 0.447	0.003	4 (4–4)	4.12 ± 0.331	5 (5–5)	4.79 ± 0.415	<0.001
	Rater 2	4 (4–4)	4.18 ± 0.393	4 (4–5)	4.47 ± 0.512	0.096	4 (4–4.75)	4.25 ± 0.447	5 (5–5)	5.00	<0.001	4 (4–4)	4.21 ± 0.415	5 (4–5)	4.73 ± 0.453	<0.001
	Rater 3	4 (4–4)	4.06 ± 0.243	5 (5–5)	4.82 ± 0.393	<0.001	4 (3.25–4)	3.75 ± 0.447	4 (4–5)	4.31 ± 0.602	0.007	4 (3–4)	3.70 ± 0.467	4 (4–5)	4.30 ± 0.529	<0.001
	Rater 4	4 (3–4)	3.65 ± 0.493	4 (4–5)	4.29 ± 0.470	<0.001	4 (4–4)	4.13 ± 0.342	5 (4–5)	4.69 ± 0.479	0.003	4 (4–4)	4.09 ± 0.292	5 (4.5–5)	4.76 ± 0.435	<0.001
	All Raters	4 (4–4)	3.99 ± 0.405	5 (4–5)	4.60 ± 0.493	<0.001	4 (4–4)	4.08 ± 0.447	5 (4–5)	4.69 ± 0.500	<0.001	4 (4–4)	4.03 ± 0.429	5 (4–5)	4.64 ± 0.496	<0.001
Visualization of the resection cavity	Rater 1	4 (4–4)	4.06 ± 0.556	5 (5–5)	4.82 ± 0.393	<0.001	4 (4–5)	4.19 ± 0.750	4 (4–5)	4.31 ± 0.704	0.414	4 (4–5)	4.12 ± 0.650	5 (4–5)	4.58 ± 0.614	0.001
	Rater 2	4 (4–5)	4.29 ± 0.470	5 (4–5)	4.59 ± 0.507	0.025	4 (4–4.75)	4.25 ± 0.447	5 (5–5)	4.94 ± 0.250	<0.001	4 (4–5)	4.21 ± 0.452	5 (4.5–5)	4.76 ± 0.435	<0.001
	Rater 3	4 (3–4)	3.65 ± 0.606	4 (4–4)	3.12 ± 0.485	0.005	4 (3–4)	3.69 ± 0.479	4 (4–4)	4.00 ± 0.632	0.059	4 (3–4)	3.67 ± 0.540	4 (4–4)	4.06 ± 0.556	<0.001
	Rater 4	4 (4–4)	4.06 ± 0.556	5 (4.5–5)	4.76 ± 0.437	<0.001	4 (3–5)	4.06 ± 0.854	4 (4–5)	4.19 ± 0.750	0.157	4 (4–5)	4.06 ± 0.704	4 (5–5)	4.48 ± 0.667	<0.001
	All Raters	4 (4–4)	4.01 ± 0.586	5 (4–5)	4.57 ± 0.527	<0.001	4 (4–4.75)	4.05 ± 0.677	4 (4–5)	4.36 ± 0.698	<0.001	4 (4–4)	4.03 ± 0.629	5 (4–5)	4.47 ± 0.623	<0.001
Diagnostic confidence	Rater 1	4 (4–5)	4.41 ± 0.507	5 (4.5–5)	4.76 ± 0.437	0.014	4 (4–4.75)	4.13 ± 0.619	5 (4–5)	4.56 ± 0.629	0.008	4 (4–5)	4.27 ± 0.574	5 (4–5)	4.67 ± 0.540	<0.001
	Rater 2	4 (4–5)	4.35 ± 0.493	4 (4–5)	4.47 ± 0.514	0.157	4 (4–4.75)	4.25 ± 0.447	5 (5–5)	4.94 ± 0.250	<0.001	4 (4–5)	4.30 ± 0.467	5 (4–5)	4.70 ± 0.467	<0.001
	Rater 3	4 (3–4)	3.59 ± 0.618	4 (4–4)	4.00 ± 0.500	0.008	4 (3.2–-4)	3.75 ± 0.447	4 (4–4.75)	4.06 ± 0.680	0.025	4 (3–4)	3.67 ± 0.540	4 (4–4)	4.03 ± 0.585	<0.001
	Rater 4	4 (4–5)	4.41 ± 0.507	5 (4–5)	4.71 ± 0.470	0.025	4 (4–4)	4.06 ± 0.574	5 (4–5)	4.50 ± 0.632	0.008	4 (4–5)	4.24 ± 0.561	5 (4–5)	4.61 ± 0.556	<0.001
	All Raters	4 (4–5)	4.19 ± 0.629	5 (4–5)	4.49 ± 0.560	<0.001	4 (4–4)	4.05 ± 0.547	5 (4–5)	4.52 ± 0.642	<0.001	4 (4–4)	4.12 ± 0.593	5 (4–5)	4.50 ± 0.599	<0.001

CR = conventional reconstruction; DLR = deep learning reconstruction; Mdn = median; IQR = interquartile range; M = mean; SD = standard deviation; T1 = T1-weighted images; CE = contrast-enhanced; and T = Tesla.

**Table 8:** Mean (range) interrater intraprotocol agreement using Kendall’s W.

	Kendall’s W		
	1.5 T (*n* = 17)	3 T (*n* = 16)	Pooled (*n* = 33)
Overall image quality_CR_	0.225 (0.131–0.331)	0.276 (0.126–0.360)	0.238 (0.128–0.304)
FLAIR_CR_	0.133	0.126	0.128
T2_CR_	0.131	0.340	0.222
T1_CR_	0.303	0.360	0.298
T1CE_CR_	0.331	0.279	0.304
Overall image quality_DLR_	0.231 (0.136–0.349)	0.413 (0.135–0.605)	0.264 (0.188–0.349)
FLAIR_DLR_	0.277	0.135	0.188
T2_DRL_	0.162	0.605	0.349
T1_DLR_	0.136	0.587	0.293
T1CE_DLR_	0.349	0.325	0.226
Visualization of the resection cavity_CR_	0.271 (0.150–0.362)	0.303 (0.173–0.546)	0.262 (0.228–0.359)
FLAIR_CR_	0.150	0.303	0.222
T2_CR_	0.272	0.546	0.359
T1_CR_	0.298	0.190	0.228
T1CE_CR_	0.362	0.173	0.237
Visualization of the resection cavity_DLR_	0.280 (0.167–0.393)	0.561 (0.484–0.730)	0.329 (0.274–0.366)
FLAIR_DLR_	0.248	0.502	0.359
T2_DRL_	0.167	0.730	0.366
T1_DLR_	0.310	0.484	0.274
T1CE_DLR_	0.393	0.527	0.315
Diagnostic confidence_CR_	0.463 (0.368–0.560)	0.463 (0.193–0.518)	0.364 (0.313–0.405)
FLAIR_CR_	0.416	0.453	0.405
T2_CR_	0.368	0.518	0.360
T1_CR_	0.509	0.193	0.313
T1CE_CR_	0.560	0.197	0.377
Diagnostic confidence_DLR_	0.361 (0.324–0.469)	0.469 (0.394–0.576)	0.354 (0.287–0.403)
FLAIR_DLR_	0.366	0.495	0.403
T2_DRL_	0.324	0.394	0.287
T1_DLR_	0.284	0.576	0.365
T1CE_DLR_	0.469	0.408	0.362

T = Tesla; FLAIR = fluid-attenuated inversion recovery; CR = conventional reconstruction; DLR = deep learning reconstruction; T2 = T2-weighted images; T1 = T1-weighted images; and CE = contrast-enhanced.

**Table 9:** Results of image quality metric (IQM) analysis between conventionally reconstructed and deep learning reconstructed MRI sequences.

Sequence	IQM	1.5 T (*n* = 17) Value (M ± SD)	3 T (*n* = 16) Value (M ± SD)	Pooled (*n* = 33) Value (M ± SD)
FLAIR	SSIM	0.9987 ± 0.0006	0.9989 ± 0.0006	0.9988 ± 0.0006
	MS-SSIM	0.9999 ± 0.0001	0.9999 ± 0.0001	0.9999 ± 0.0001
	FSIM	0.8420 ± 0.0316	0.8242 ± 0.0278	0.8337± 0.0298
	NQM	14.3721 ± 2.1645	15.0680 ± 1.9378	14.6983 ± 2.0582
	SNR	13.6127 ± 2.0214	15.0649 ± 2.2866	14.2934 ± 2.1457
	PSNR	67.2675 ± 3.2487	67.8192 ± 2.6083	67.5261± 2.9485
T2	SSIM	0.9965 ± 0.0029	0.9924 ± 0.0035	0.9947 ± 0.0032
	MS-SSIM	0.9995 ± 0.0004	0.9985 ± 0.0008	0.9991 ± 0.0006
	FSIM	0.8159 ± 0.0428	0.8440 ± 0.0458	0.8284 ± 0.0441
	NQM	14,1331 ± 2.8435	14.2798 ± 2.7657	14.2008 ± 2.8076
	SNR	13.8342 ± 2.2077	12.9179 ± 2.0395	13.4269 ± 2.1329
	PSNR	61.1301 ± 3.1127	56.6777 ± 2.5528	59.1513 ± 2.8639
T1	SSIM	0.9801 ± 0.0078	0.9982 ± 0.0010	0.9882 ± 0.0047
	MS-SSIM	0.9978 ± 0.0011	0.9997 ± 0.0002	0.9986 ± 0.0007
	FSIM	0.6860 ± 0.0763	0.8475 ± 0.0298	0.7584 ± 0.0555
	NQM	4.2984 ± 2.7396	15.9673 ± 2.2263	9.5293 ± 2.5095
	SNR	6.0580 ± 1.6684	14.9067 ± 1.8536	10.0246 ± 1.7514
	PSNR	55.3725 ± 3.7210	63.4592 ± 3.9854	58.9975 ± 3.8395
T1CE	SSIM	0.9726 ± 0.0090	0.9957 ± 0.0028	0.9838 ± 0.0060
	MS-SSIM	0.9967 ± 0.0013	0.9994 ± 0.0004	0.9980 ± 0.0009
	FSIM	0.6599 ± 0.0685	0.8311 ± 0.0322	0.7427 ± 0.0509
	NQM	3.1430 ± 2.1562	15.6468 ± 2.1087	9.1932 ± 2.1332
	SNR	5.2700 ± 1.4386	14.9875 ± 1.8921	9.9720 ± 1.6580
	PSNR	52.6580 ± 3.0930	60.9097 ± 3.8099	56.6507 ± 3.4399

Data are presented as mean ± standard deviation. IQM = image quality metric; T = Tesla; M = mean; SD = Standard Deviation; SSIM = Structural Similarity Index; MS-SSIM = Multi-Scale SSIM; FSIM = Feature Similarity Index; NQM = Noise Quality Metric; SNR = signal-to-noise ratio; and PSNR = peak signal-to-noise ratio.

**Table 10:** Detection rate in absolute number of residual tumor following resection of intracranial tumors on 1.5 T (*n* = 17) and 3 T (*n* = 16) between DLR and CR protocols.

		DLR > CR	DLR = CR	DLR < CR
Lesion_1.5T_ (*n* = 17)	Rater 1	0	11/17	0
	Rater 2	4/17	6/17	0
	Rater 3	1/17	6/17	0
	Rater 4	2/17	9/17	0
Lesion_3T_ (*n* = 16)	Rater 1	0	7/16	0
	Rater 2	1/16	10/16	0
	Rater 3	0	3/16	0
	Rater 4	0	6/16	0
Lesion_pooled_ (*n* = 33)	Rater 1	0	18/33	0
	Rater 2	5/33	16/33	0
	Rater 3	1/33	9/33	0
	Rater 4	2/33	15/33	0

DLR > CR, DLR protocol detects more lesions compared to CR protocol; DLR = CR, residual tumor tissue was detected in both DLR and CR protocols; DLR < CR, CR detects more lesions compared to DLR protocol. T = Tesla; CR = conventional reconstruction; DLR = deep learning reconstruction.

## Data Availability

In order to safeguard the confidentiality of the participants, the data pertaining to this study are currently withheld from public access. The data can be shared upon request.

## Supplementary Materials

The following supporting information can be downloaded at https://www.mdpi.com/article/10.3390/diagnostics15151982/s1, Figure S1: Individual visual preferences in the review of paired magnetic resonance image sets following tumor resection at 1.5 T (A) and 3 T (B) by four experienced raters. Raters consist of a neuroradiologist (Rater 1), a neurosurgeon (Rater 2), a neuro-oncologist (Rater 3), and a radiation oncologist (Rater 4). CR = conventional reconstruction; DLR = deep learning reconstructed technique; FLAIR = fluid-attenuated inversion recovery images; T2 = T2-weighted images; T1 = T1-weighted images. CE = contrast-enhanced; and T = Tesla.

## Abbreviations

The following abbreviations are used in this manuscript:

CNR	Contrast-to-noise Ratio
CR	Conventional Reconstruction
DL	Deep Learning
DLR	Deep Learning-based Reconstruction
FDA	U.S. Food and Drug Administration
FLAIR	Fluid-attenuated Inversion Recovery
FSIM	Feature Similarity Index
MS-SSIM	Multi-Scale SSIM
NQM	Noise Quality Metric
PAT	Parallel Acquisition Technique
PSNR	Peak SNR
SD	Standard Deviation
SE	Spin Echo
SNR	Signal-to-noise Ratio
SSIM	Structural Similarity Index
TSE	Turbo Spin Echo
