# Supplementary figures and images for: Multidisciplinary, Clinical Assessment of Accelerated Deep-Learning MRI Protocols at 1.5 T and 3 T After Intracranial Tumor Surgery and Their Influence on Residual Tumor Perception
Source: Diagnostics (Basel). 2025 Aug 7;15(15):1982. doi: 10.3390/diagnostics15151982 (PMC12346804; doi:10.3390/diagnostics15151982)

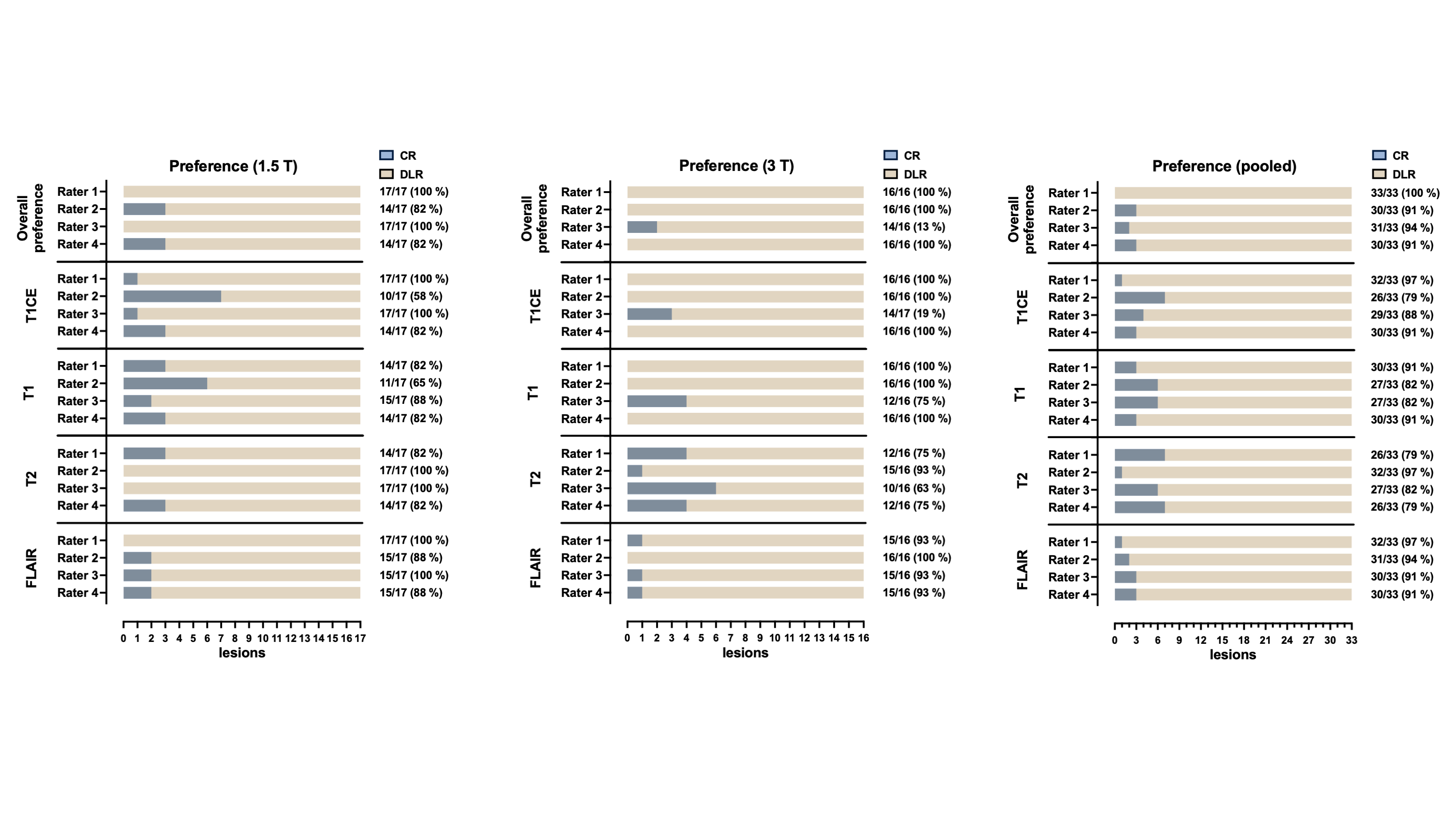

Supplement: Supplementary file 1 [file diagnostics-15-01982-s001.zip › diagnostics-3665359-supplementary.tiff]
